# Cardiometabolic Health Status, Ethnicity and Health-Related Quality of Life (HRQoL) Disparities in an Adult Population: NutrIMDEA Observational Web-Based Study

**DOI:** 10.3390/ijerph19052948

**Published:** 2022-03-03

**Authors:** Rosa Ribot-Rodriguez, Andrea Higuera-Gomez, Rodrigo San-Cristobal, Roberto Martín-Hernández, Víctor Micó, Isabel Espinosa-Salinas, Ana Ramírez de Molina, J. Alfredo Martínez

**Affiliations:** 1 Precision Nutrition and Cardiometabolic Health, IMDEA-Food Institute (Madrid Institute for Advanced Studies), Campus of International Excellence (CEI) UAM+CSIC, 28049 Madrid, Spain; rosa.rib.rod@gmail.com (R.R.-R.); andrea.hg92@gmail.com (A.H.-G.); roberto.martin@imdea.org (R.M.-H.); victor.mico@imdea.org (V.M.); jalfredo.martinez@imdea.org (J.A.M.); 2 Bioinformatics and Biostatistics Unit, Madrid Institute for Advanced Studies IMDEA Food, CEI UAM+CSIC, 28049 Madrid, Spain; 3 Nutritional Genomics and Health Unit, Research Institute on Food and Health Sciences IMDEA Food, UAM+CSIC, 28049 Madrid, Spain; mariaisabel.espinosa@imdea.org; 4 Molecular Oncology Group, Research Institute on Food and Health Sciences IMDEA Food, UAM+CSIC, 28049 Madrid, Spain; ana.ramirez@imdea.org; 5 CIBERobn Physiopathology of Obesity and Nutrition, Institute of Health Carlos III (ISCIII), 28029 Madrid, Spain

**Keywords:** precision nutrition, web-based health surveys, HRQoL, nutrimeter, public health

## Abstract

Precision public health supported on online tools is increasingly emerging as a potential strategy to achieve health promotion and disease prevention. Our aim was to assess the relationships of sociodemographic variables, anthropometric data, dietary habits and lifestyle factors with health-related quality of life (HRQoL), cardiometabolic health status and ethnicity in an online recruited adult population (NutrIMDEA Study). NutrIMDEA Study is a web-based cross-sectional survey that included 17,333 adults. Self-reported sociodemographic characteristics, anthropometric data, clinical and family history of cardiometabolic illnesses, dietary habits, lifestyle factors and HRQoL features were collected. Diseased individuals showed significative poorer MedDiet and worse HRQoL than those in the healthy cardiometabolic status group (*p* < 0.05). In comparison, European/Caucasian individuals reported a significantly better HRQoL, higher MedDiet and HRQoL values compared with those of other ethnicities (*p* < 0.05). We obtained a total of 16.8% who reported poor/fair, 56.5% good and 26.6% very good/excellent HRQoL. Respondents with very good/excellent HRQoL showed lower BMI, greater adherence to a Mediterranean diet (MedDiet) and higher physical activity. The results suggest the presence of interactions between the mental and physical components of HRQoL with obesity, sedentarism and dietary intake, which were dependent on disease status and ethnicity. Online HRQoL assessment could contribute to wider implementation of precision public health strategies to promote health targeted interventions with policy implications to community health promotion.

## 1. Introduction

Public Health Nutrition (PHN) involves the study of the environment, sociodemographic characteristics, diet, lifestyle and health, which affects the design, implementation and evaluation of nutritional interventions at the community level in order to improve the health status of specific groups [1,2]. In the new era of availability of information and big data, the Precision Public Health (PPH) concept is emerging, based on applying “the right intervention at the right time, every time to the right population”. In this way, considering health-related factors as possible, better target interventions and policies for populations can be accounted for, which includes considerations of social and environmental determinants of health [3]. In this context, accompanying cardiometabolic complications (obesity, diabetes, hypertension and dyslipidemia) are closely related to eating and metabolic status and are a focus of multiethnic and intersectoral actions in public health nutrition, which needs to be analyzed and actions implemented based on personalized information [4], where online data sourcing will be the way for future investigations [5].

In this context, health indicators are defined as summary measures that capture relevant information on different attributes and dimensions of the state of health and performance [6]. These estimators help to screen and monitor the health of a particular population [7]. The traits include the characterization of quality of life, where the dimensions of health include physical, emotional, spiritual, environmental, mental and social well-being [8]. Some of these meters and indicators are metabolic status assessment, health-related quality of life (HRQoL), prevalence of noncommunicable diseases (NCD) and determinants of health and lifestyle (dietary patterns, physical activity or smoking status) factors, which may be retrieved by online surveys [9].

Furthermore, health-related quality of life (HRQoL) is a measure of the impact of health/disease on daily functions and is greatly influenced by an individual’s conditions, concerns and aspirations as well as self-perceived health and well-being [10]. Determinants of health quality and alleged well-being include sociodemographic, environmental and nutritional features such as diet and lifestyle factors [11]. There is a wide range of instruments for measuring HRQoL such as Short-Form 36 (SF-36), Short-Form 12 (SF-12), European Quality of Life-5 Dimensions (EQ-5D), World Health Organization Quality-of-Life Scale (WHOQoL) and Nottingham questionnaire [12] among others [13]. Today, the SF-36 questionnaire is one of the most worldwide-administered tools to evaluate the multidimensional HRQoL The SF-12 Spanish validated version is made up of a subset of twelve items of the SF-36 including one or two questions of each of the eight scales of the SF-36 [14]. The information from these twelve items is used to construct the physical and mental summary measures of the SF-12 (PCS12 and MCS12, respectively), both of them representing specific global health dimensions [15].

The epidemiological rates concerning cardiometabolic-related morbidities (obesity, diabetes, hypertension and dyslipidemia) have increased in recent years, with a growing recognition by the population that PH policies and modifiable factors affect health and disease outcomes [16] through the life course [17]. In contrast, a number of studies have separately investigated disease and HRQoL relationships [18] and trends of NCD depending on race/ethnicity [19], but there are fewer studies that associate genetic/social factors with HRQoL, instead suggesting the existence of risk populations [20]. Newer studies today should be devoted to determining the prevalence of major diseases and risk factors concerning migrants and racial groups to guide health policies concerning wellbeing in vulnerable ethnicities with precision [19,20].

In this framework, a precision medicine and nutrition approach involving PPH perspectives is important to improve quality of life, diet-related habits and healthy lifestyles to reduce the risk of future cardiometabolic diseases considering health and ethnic aspects. To this end, the aim of the current study is the assessment of relationships among sociodemographic variables, cardiometabolic diseases/morbidities (obesity, diabetes, hypertension and dyslipidemia), dietary habits and lifestyle factors as well as putative interaction with health-related quality of life, where the role of ethnicity on some analyzed outcomes was examined in an online recruited population.

## 2. Materials and Methods

### 2.1. Study Design and Sample

Survey data were collected from the web-based NutrIMDEA Study. A total of 17,333 participants (62.7% females and 37.3% males) were included between May 2020 and November 2020 in this observational cross-sectional study. Inclusion criteria considered to enroll participants with age over 18 years with internet access, with the only requirement that they understood Spanish to complete the survey and not necessarily being from a Spanish-speaking country. The questionnaire was freely online accessible at https://nutrimdea2020.questionpro.com/ (accessed on 8 July 2020). Sources of information were an open survey and a rewarded survey. The first one was advertised in different communication/media channels as Spanish national newspapers or radio programs, and in the second one, audiences were purchased by omnibus companies in Spanish-speaking countries. Responders that completed the open survey obtained a personalized report based on their habits and health. Self-reported answers from multiethnic participants from Spanish-speaking countries were analyzed, where the Spanish National Health Survey 2017 (SNHS 2017) was used as reference for comparisons [21]. All those individuals who showed interest to be part of the study were properly informed about all the procedures before they entered the study. The questionnaire was delivered after asking conditions to IMDEA-CEI and the external companies that performed the surveys, which confirmed that filling the questionnaires is a proof of acceptance to participate and contribute to the NutrIMDEA study with own anonymized data. In our case, a disclaimer was incorporated to the survey to inform about these matters.

### 2.2. Questionnaire and Measurements

The survey was based on the questionnaire of de Cuevillas et al. [22], where quality of life phenotypical and lifestyle factors (diet/physical activity) were recorded to categorize individuals with a nutritional quantitative score or nutrimeter according to their nutritional well-being in order to discriminate nutritypes.

The baseline questionnaire included sociodemographic data (age, sex and educational level/occupation), self-reported anthropometric data (weight and height), cardiometabolic diseases prevalence (obesity, diabetes, hypertension, dyslipidemia), family history of cardiometabolic diseases (obesity, diabetes, hypertension, dyslipidemia), dietary habits (Mediterranean Adherence Score, number of meals per day, snacking habit, servings of vegetables per day, servings of legumes per day, servings of fish per day), lifestyle (nap habit, physical activity, smoking status) and quality of life features (SF12 Health Survey Item 1) or calculated physical and mental component scores of SF12 Health Survey (PCS12/MCS12). All of these variables were self-reported by the participants or calculated from the responses.

Among sociodemographic data, the age was analyzed within three categories (18–40 years, 40–70 years and >70 years). Sex consisted of two categories (male and female), while the category “other” was avoided in the current analyses. Educational level was classified into two categories: high school or less (primary education or less, low or intermediate secondary education, higher secondary education) and more than high school (intermediate vocational education, higher vocational education or university). Occupation was defined in three main groups: unemployed/retired, worker and student.

Concerning anthropometric data, BMI was calculated using data on self-declared weight and height, and individuals were stratified according to their Body Mass Index (BMI). Cut-off points were established according to the World Health Organization (WHO) as normal weight (BMI < 25.0 kg/m^2^), overweight (BMI 25.0–29.9 kg/m^2^) or obesity BMI ≥ 30.0 kg/m^2^) [23].

Cardiometabolic disease prevalence was self-reported by participants with the following question: “Have you been diagnosed or are you currently undergoing treatment for any of the following conditions?” The options were diabetes, high blood pressure, dyslipidemia and obesity, and possible answers were yes/no. 

As nutritional quality estimation, the Mediterranean Adherence Diet Score was assessed by using the PREDIMED questionnaire, known as MEDAS-14 [24]. In addition, dietary habits such as number of meals a day, snacking habit and servings of vegetables, legumes and fish per week were recorded. In relation to lifestyle, nap habit, which is defined as a short period of sleep typically taken during daytime hours as an adjunct to the usual nocturnal sleep period, was categorized as yes/no. Physical activity was assessed using the International Physical Activity Questionnaire (IPAQ) using the Spanish version [25]. Activities were categorized as light, moderate or intense based on the metabolic equivalent value (MET), which was converted to hours/week units. Smoking status was categorized as current or former smoker.

Quality of Life Features were assessed with Item 1 of SF12 Health Survey (In general, would you say your health is?), and physical and mental component scores of SF12 Health Survey (PCS12/MCS12), were computed, which ranged from 0 to 100. High scores indicated a better quality of life [26]. Participants were categorized into three groups (poor/fair health, good health and very good/excellent health) according to self-reported answer SF12 Health Survey Item 1 “In general, would you say your health is?” This item was transformed: poor and fair were pooled into the group of “poor/fair” HRQoL, good into the group “good” HRQoL and very good and excellent made up the “very good/excellent” group, a third category. Moreover, participants were stratified by cardiometabolic health status into two groups (healthy cardiometabolic status and diseased cardiometabolic status). A diseased group was assigned if a participant had one of these cardiometabolic diseases (obesity, diabetes, hypertension or dyslipidemia). The last stratification was performed by ethnicity into two groups (European/Caucasians and other ethnicities). Other ethnicities included Africans, Asians, Hispanic/Latinos, mestizos and other ethnicities. 

### 2.3. Statistical Analysis

For the Spanish population, the survey showed a margin of error considered as the degree of error in results received from random sampling surveys [27,28] of 0.9% with a confidence level of 95% (46,940,000 population and 11,883 sample). For the Hispanic sample, we took into account the countries of those with +20 observations. We calculated the same 0.9% margin of error with a 95% confidence level (1,055,910,000 population and 11,883 sample).

Characteristics of the study sample were presented using descriptive statistics such as mean and standard deviation for continuous variables or proportions for categorical variables. Differences in sociodemographic data, dietary patterns and lifestyle features according to HRQoL, cardiometabolic health status and ethnicity were assessed using either chi-squared tests (χ^2^ test), two-sided student’s *t*-tests or one-way analysis of variance (ANOVA). Significance threshold for the obtained *p*-values was set to *p* < 0.05. All descriptive and statistical analyses were performed using the R programming software (version 3.6.0; R Foundation (RStudio, PBC, Boston, MA, USA)). 

## 3. Results

### Sample Characteristics

Participants (*n* = 17,333) of the NutrIMDEA 2020 Study (Table 1) were mainly females (62.7%) and had a higher educational level than the ones included in the Spanish National Health Survey 2017 (SNHS 2017). Our study population had better cardiometabolic health with less prevalence of obesity, HBP, diabetes and dyslipidemia, fewer current smokers, better self-perception of their health, lower BMI, greater consumption of vegetables per day and no significant differences in light physical activity. The SNHS 2017 population was older, with 22% of participants over 70 years of age compared to 5% of this age range in the NutrIMDEA Study. Regarding occupation, the large percentage of retirees in the SNHS 2017 is also noteworthy, and in terms of education level, NutrIMDEA mostly reaches university studies.

Results after categorizing the sample by cardiometabolic health status are reported (Table 2). The diseased cardiometabolic group had at least one of the following cardiometabolic diseases: obesity, diabetes, hypertension or dyslipidemia. There were significant differences in all variables except for snaking habit, servings of legumes per day and moderate/intense and total physical activity. The analyzed sample had mostly healthy cardiometabolic status (71%), and the majority age group in diseased participants was 40–70 years and included more diseased women than men. Most of the sample was university collective and had a higher percentage of workers among the healthy participants (75.4% vs. 68.8%). The diseased group (29%) showed more family history of cardiometabolic diseases, included more current smokers and presented a worse HRQoL than the healthy group (Table 2). PCS12 in the healthy group was higher (54.5 (95% CI, 54.4–54.6) points vs. 51.0 (95% CI, 50.7–51.23) points with *p* < 0.05). Surprisingly, MCS12 was higher in the diseased group (44.7 (95% CI, 44.3–45.0) points vs. 43.5 (95% CI, 43.3–43.7) points with *p* < 0.05).

**Figure 1:**
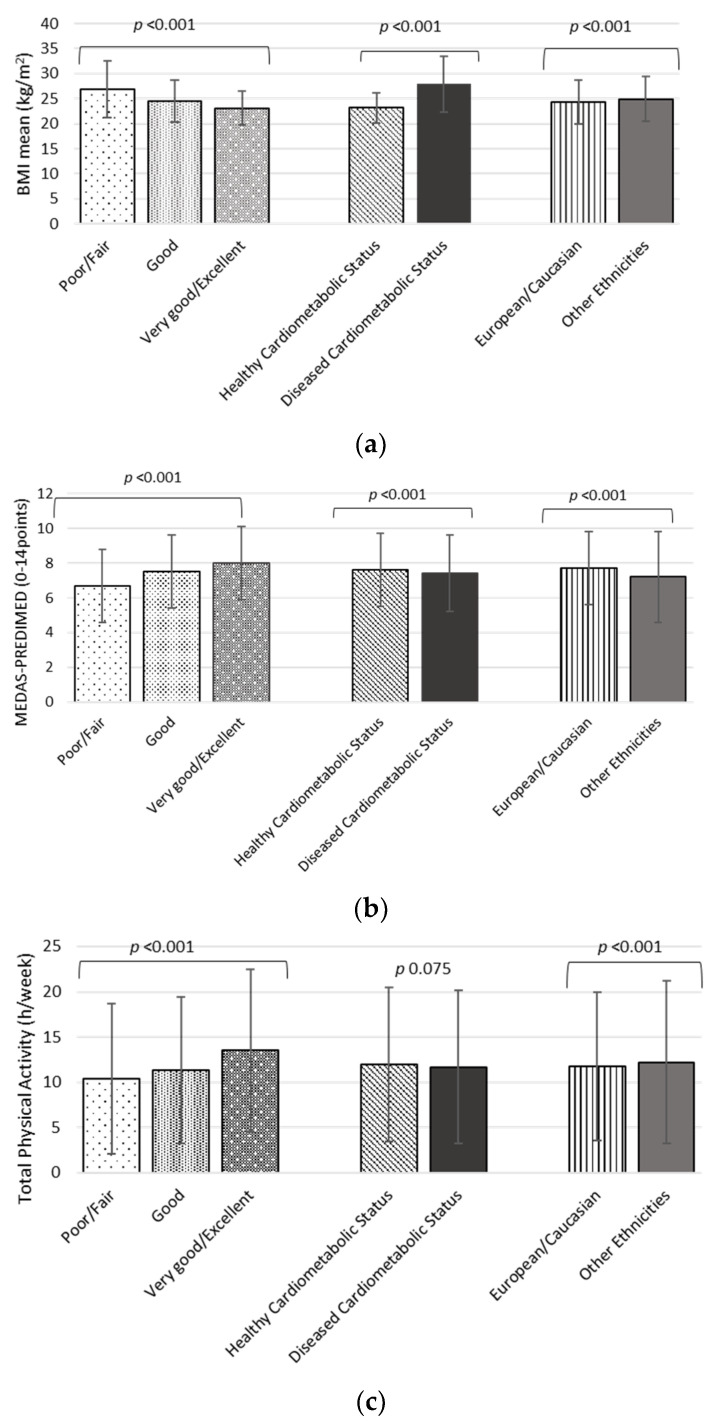
Descriptive Health Characteristics and Lifestyle Categorized by HRQoL, Cardiometabolic Health Status and Ethnicity of the participants of the study. (**a**) BMI mean Categorized by HRQoL, Cardiometabolic Health Status and Ethnicity; (**b**) Mediterranean Adherence Score (MEDAS)–PREDIMED Categorized by HRQoL, Cardiometabolic Health Status and Ethnicity; (**c**) Total Physical Activity Categorized by HRQoL, Cardiometabolic Health Status and Ethnicity.

**Figure 2:**
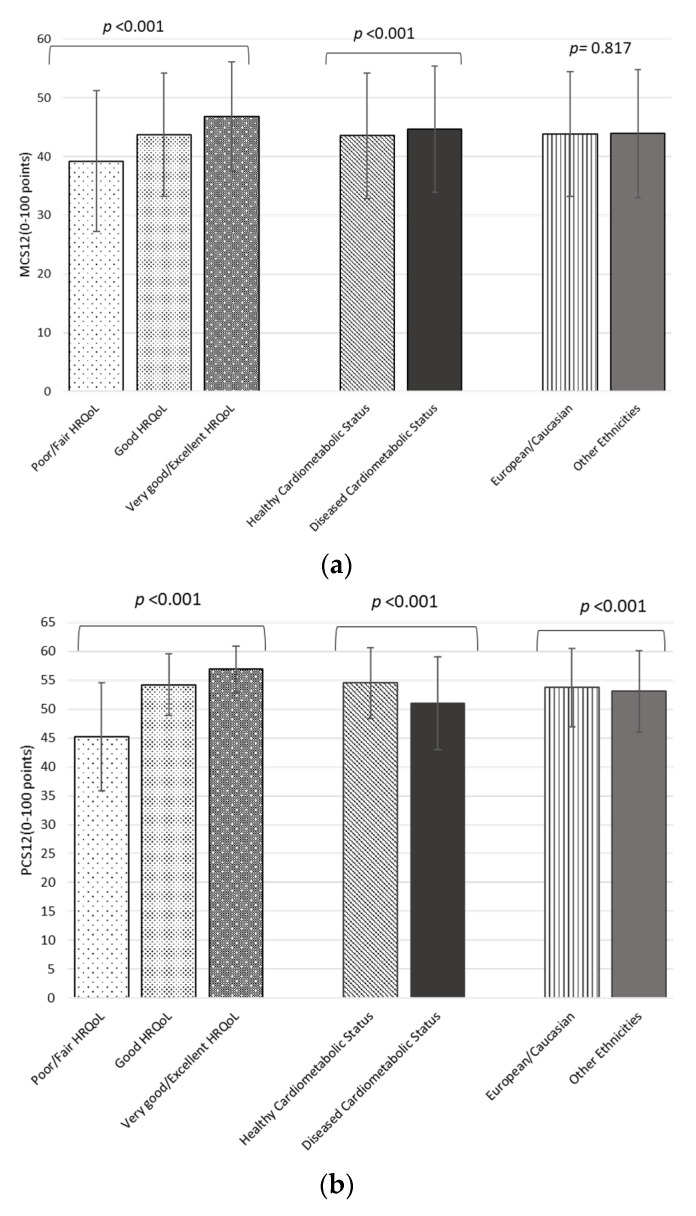
Mental (**a**) and Physical (**b**) Component Summary Score of SF-12 Health Survey Categorized by Cardiometabolic Health Status and Ethnicity.

The main characteristics of the participants according to their ethnicity (European/Caucasian and other ethnicities) are also displayed (Table 3). There were no significant differences in diabetes prevalence, family history of HBP, light physical activity and MCS12. European/Caucasian individuals included in the study were younger compared to other ethnicities, less obese (9.9% vs. 12.3% with *p* < 0.05), were less likely to be current smokers and had less family history of obesity and family history of dyslipidemia compared to other groups. Europeans/Caucasian reported eating more meals a day and having less snacking habit than other ethnicities. BMI mean and Total PA was higher in other ethnicities and showed significant differences with Europeans/Caucasians (Figure 1), while high rates of MEDAS-14 were achieved in such group. A higher PCS12 for the European group (53.7 (95% CI, 53.6–53.9) points vs. 53.0 (95% CI, 52.8–53.2) points, *p* < 0.05) was found, while no significant differences in MCS12 (43.9 (95% CI, 53.6–44.1) points in both groups, *p* = 0.817) where noted (Figure 2). 

Regarding HRQoL, 16.8% of the sample of NutrIMDEA Study reported a poor/fair HRQoL, 56.5% of the surveyed subjects reported good self-perception of health and 26.6% reported a very good/excellent HRQoL (Table 4). There were significant differences in all variables when they were categorized by Item 1 of SF12 Health Survey. The participants who reported a very good/excellent HRQoL were mostly 40–70 years old, had a higher educational level, were workers and had a lower prevalence of cardiometabolic diseases and less family history of cardiometabolic diseases. A higher percentage of responders reported very good/excellent HRQoL, never smoked and showed higher scores in MCS12 (46.8 (95% CI, 46.5–47.1) points vs. 39.2 (95% CI, 38.7–39.7) points *p* < 0.05) and PCS12 (56.9 (95% CI, 56.8–57.1) points vs. 45.2 (95% CI, 44.8–46.5) points in poor/fair HRQoL, *p* < 0.05). Moreover, they reported lower weight, less obesity, more consumption of vegetables per day, more servings of legumes per day and more total physical activity than poor/fair HRQoL responders.

Global trends of main inputs and outcomes are illustrated (Figure 1). Thus, in HRQoL, there is a negative association with BMI and a positive association with MEDAS14 and total physical activity. Regarding diseased cardiometabolic status, it was established a direct association with BMI and a negative association with MEDAS14 and total physical activity, but in this last variable with statistically marginal differences (12.0 (95% CI, 11.4–12.1) hours/week vs. 11.7 (95% CI, 11.8–12.2) hours/week, *p* = 0.075). Regarding ethnic issues, other ethnicities showed a positive association with BMI, a negative association with MEDAS-14 and higher total physical activity than European/Caucasian respondents.

As a measure of global health, MCS12 and PCS12 were assessed (Figure 2). MCS12 showed significative differences between the three categories HRQoL (poor/fair, good and very good/excellent) (39.2 ± 12.0 (95% CI, 38.7–39.7) points, 43.7 ± 10.5 (95% CI, 43.5–44.0) points and 46.8 ± 9.3 (95% CI, 46.5–47.1) points, respectively, with *p* < 0.001). Furthermore, there was a remarkable difference in MCS12 between either healthy or diseased cardiometabolic status (43.5 ± 10.7 (95% CI, 43.3–43.7) points and 44.7 ± 10.7 (95% CI 44.3–45.0) points, respectively, *p* < 0.001). However, there were no statistical differences in HRQoL according to ethnicity (*p* = 0.817). Noteworthily, PCS12 showed differences among all stratifications in the applied statistical tests.

## 4. Discussion

Public health strategies have considered nation-wide surveys as NHANES (National Health and Nutrition Examination Survey) to assess the health and nutritional status of adults and children in the United States [29] or EHIS (European Health Interview Survey) that aims at measuring on a harmonized basis and comparability among Member States the health status, health determinants and access to health of the EU citizens [30]. In Spain, the Spanish National Health Survey (SNHS) is carried out by the Spanish Ministry of Health, Consumption and Social Welfare with the collaboration of the Spanish National Institute of Statistics (INE) and collects health information related to the resident population in Spain [21]. This approach is a five-year study that allows researchers to know health aspects from citizens at national and regional levels by collecting data to plan, implement and evaluate actions in health matters, which was used to analyze our web-based population as the closest customary standard.

In this sense, we compared the latest SNHS 2017 reference population survey with the NutrIMDEA Study sample, aiming to find potential similarities/differences on several health indicators and health determinants in order to contextualize the health situation of the study population based on two different models. A wider proportion from the SNHS 2017 participants were older than 70 years, with a higher percentage of retirees compared to the NutrIMDEA Study, which was not unexpected given the data origin: traditional collection method vs. online, respectively. Accordingly, as a consequence of age distribution, most variables evidenced better health outcomes in the NutrIMDEA Study. This fact may be also be because the NutrIMDEA Study, as an online directed health survey, may imply that the participants are more aware and interested in health-related issues, as seen in previous web-based studies such as Food4Me [31]. In any case, the online health data collection method has been validated [31], where important variables such as reported and collected BMI are correlated [32]. In this direction, we can confirm that SNHS 2017 and NutrIMDEA Study involve noncomparable populations due to the data collection method, different periods of data inclusion (prior to 2017 and in 2020, respectively) and assumably different internet knowledge in an older Spanish population, which may explain the outcome heterogeneity and some input diversity.

Sociodemographic features, dietary habits and lifestyle factors are related to health and quality of life [33]. Our population showed expected trends concerning the categories sex, education, occupation, anthropometric data and lifestyle (nap habits, physical activity and smoking status), as previously published in comparable populations [11,34] Participants who reported a high self-referred HRQoL value showed better health outcomes, as found in previous studies in Spain [35,36]. Thus, dietary habits were more balanced as HRQoL increases and higher consumption of vegetables and fish were associated with superior HRQoL as found in a previous study by Sayón-Orea et al. [37]. Moreover, obesity was significantly related to a lower HRQoL [38]. There is an increasing trend of PCS12 and MC12 values as the HRQoL category rises. This direction also remains in the MCS12 value on both healthy/diseased groups. However, this tendency is reversed in the PCS12 score, which could be explained by a reduced physical activity. There were no significant differences in MCS12 between European/Caucasians and other ethnicities. An independent effect of BMI on HRQoL among racial and ethnic groups was previously identified in an investigation involving Blacks, Whites and Hispanics [39]. 

Cardiometabolic health status depends on sociodemographic characteristics, dietary habits and lifestyle [11,40]. In this sense, metabolic syndrome has been associated with poor overall health and poor physical mental health status in American adults [41], which is consistent with our results. Furthermore, a higher education level among the healthy group was detected, and twice, unemployed/retired were found in the diseased group, which may be due to older aged subjects in this category. Data collection has some influence since hypertension rates in the current cohort were lower than data provided by other studies [42,43]. Indeed, in an online population, it may be found that only those with antihypertension treatment may respond as suffering cardiometabolic manifestation, which also applies for the diabetes, obesity and dyslipidemia questions/answers. The higher percentage of family history of cardiometabolic diseases in the diseased group can be explained by genetic factors and worse inherited and shared domestic habits [44].

According to previous studies, normal weight participants had a lower probability to report poor HRQoL than overweight/obese participants with any chronic disease [37]. It was unexpected to not find significant differences in moderate, intense and total physical activity despite previous references in the literature [37]. Perhaps this finding may be partly due to the data collection period during the COVID-19 pandemic, which may have led changes in lifestyle.

In Europe, previous ethnicity research has reported inconsistent findings regarding health status and HRQoL [45]. Our study adds to the current evidence some multifactorial findings since more HBP prevalence in other ethnicities was found and higher normal weight prevalence in European group. On the other hand, the “Healthy migrant effect” is a situation where migrants often have a better health status than the remaining population in the native country, but not concerning BMI data, as contrasted in another study [46]. This same study showed other ethnicities had the highest odds of HRQoL, with worse health than White British adults [46]. In contrast, a higher PCS12 score in Europeans/Caucasians than in other ethnicities was found, while MCS12 was similar in both groups.

A negative correlation among very good/excellent HRQoL and high BMI was featured, which is consistent with previous research [11,47]. Moreover, a positive association among very good/excellent HRQoL with MEDAS14 was identified, which is in agreement with data from the PREDIMED-PLUS trial, where MEDAS14 has been associated with better self-reported quality of life and attributed to the fact that Mediterranean diet reduces the risk of chronic diseases and is protective against cardiovascular diseases [48,49]. In addition, total physical activity levels are associated with better HRQoL and dietary patterns, as reported in a previous studies [50,51]. These observations confirm the suitability of a web-based collection and provide support for actions focused on improving lifestyle and control obesity in the population based on online captured information. Previous studies have noted the protective effect of a high-quality diet on self-perceived health [11,47,48] as well as similar results observed in another European cohort that showed that higher adherence to a Mediterranean diet was associated with higher HRQoL, as assessed by other means [52]. In other studies, lower HRQoL scores were reported by people who were older, with elementary education or less and with fair or poor subjective health status, factors which are affected by ethnicity [53].

An overview of systematic reviews evidenced that sedentary behaviors are related with adverse health outcomes, which can benefit from computer and internet use [54]. Furthermore, an online approach may facilitate counseling in adults without known cardiovascular disease [55]. The comparison of data from subjects with different cardiometabolic status in our cohort demonstrated the reliability of the online instrument concerning the collection of anthropometrics, dietary habits, lifestyle and quality of life data. However, despite the plausibleness of the outcomes, this cross-sectional research was not able to address the issue if such relationships are causal for disease or are a consequence of a poor cardiometabolic health.

Concomitantly, the analyses of the survey responses concerning ethnic backgrounds revealed differences in personal and phenotypical features, as previously reported in Canadian immigrants and native-born individuals [56], which may be of help to implement future culturally based interventions for chronic disease management, as also reviewed in Chinese–Americans [57]. As other research has evidenced, a sense of belonging contributes positively to subjective well-being, particularly in countries with high ethnic heterogeneity [58]. Similarly, some studies have tried to prove that Europeans have a particularly negative relationship between the sense of pride and belonging concerning the component of identification, which may play a role in more homogeneous cultures, reducing the well-being status [59].

These findings are important for research and clinical applications in adult populations, because it may help to identify at-risk populations for nutritional screening by self-reported quality of life, self-reported cardiometabolic status and by ethnicity. Moreover, findings from this study can support targeted efforts to guide precision public health and to make aware healthcare professionals. From this approach, a prediction model for risk stratification is to identify potential risk groups to screening cardiometabolic health status and analyze the information on health and nutritional status to personalize individualized nutritional advice.

### Strengths and Limitations

Some methodological considerations concerning this research are required since the self-reported online data collection comprise a free open survey and rewarded survey, which should be taken into account when interpreting the data. In any case, both web-based surveys have been proven to be useful, easy and sustainable compared with traditional presential surveys. Indeed, given the design of this study, it cannot establish causal inferences from the associations found but can generate hypothesis that could be assessed in future prospective trials. Therefore, the current results need to be interpreted with caution. The descriptive analyses included comparisons concerning age, sex and BMI to understand the role of these potential confounders in relation to quality of life, cardiometabolic status and ethnicity. Furthermore, current data were collected during the COVID-19 pandemic, so there might be several influences on habits and lifestyle and the inherent limits of prediction for individuals. The online collection and the need that participants have some web knowledge as internet users need to be also accounted for. Thus, some surveys such as the Nurses´ Health study [60] and the Health Professionals Follow-up [61] have reached useful conclusions, based on face-to-face interviews, while online collection has been validated in other surveys such as the SUN cohort [62] and Food4Me [9], supporting the validity or our findings involving an online recruited population.

A major strength of this study is the relatively large sample size, which backs web-based population representativity as well as an easy and reliable web-based data collection, where dietary behavior records can be as effective as a conventional approach [32]. Another important strength is the wide number and diversity of variables collected in the survey with validated tools that allowed a precision personalized analysis, while the important number of participants and origin diversity contribute to the representativity of the screened sample population. 

## 5. Conclusions

In conclusion, in the NutrIMDEA web-based population, we found that age, high education, high PCS12 and MCS12, high MEDAS-14 and a healthy lifestyle were positively associated with very good/excellent HRQoL. The relationships between life quality and several lifestyle modifiable factors were found to be different in healthy and diseased groups. Interestingly, analyses by ethnicity concerning online self-reported health information is a pioneer manner to study sociodemographic and quality of life interactions, which revealed inequalities and inadequacies in wellbeing depending on ethnic backgrounds.

## Figures and Tables

**Table 1:** Sociodemographic Data, Health Characteristics, Dietary Habits, Lifestyle and Quality of Life Features Categorized by Spanish National Health Survey 2017 and NUTRIMDEA Study 2020.

Characteristics	Spanish National Health Survey 2017	NUTRIMDEA Study 2020	*p*-Value
*n*	22,512	17,333	
Sociodemographic data			
Age (%)			<0.001
18–40 years	5693 (25.0)	6778 (39.0)	
40–70 years	11,860 (53.0)	9777 (56.0)	
>70 years	4958 (22.0)	777 (5.0)	
Sex (%)			<0.001
Male	10,305 (45.8)	6403 (37.0)	
Female	12,207 (54.2)	10,862 (62.7)	
Education (%)			<0.001
High school or less	15,029 (67.0)	2787 (16.1)	
More than high school	7482 (33.0)	14,545 (83.9)	
Occupation (%)			<0.001
Unemployed/retired	9665 (43.0)	3333 (19.2)	
Worker	12,071 (54.0)	12,725 (73.4)	
Student	747 (3.0)	1274 (7.4)	
Anthropometric data			
BMI Category (WHO Criteria; %)			<0.001
Underweight	995 (4.5)	633 (3.7)	
Normal weight	8913 (40.5)	10,110 (58.3)	
Overweight	8225 (37.3)	4738 (27.3)	
Obesity	3907 (17.7)	1850 (10.7)	
Cardiometabolic diseases prevalence			
Obesity (%)	2634 (11.7)	1228 (7.1)	<0.001
Diabetes (%)	2265 (10.1)	632 (3.8)	<0.001
HBP (%)	6238 (27.7)	1678 (9.7)	<0.001
Dyslipidemia (%)	5453 (24.2)	2613 (15.1)	<0.001
Dietary Habits			
Servings of vegetables per day (%)			<0.001
1 or no servings per day	4081 (18.1)	2979 (17.2)	
2 or 3 servings per day	15,817 (70.3)	10,803 (62.4)	
More than 3 servings per day	2594 (11.5)	3528 (20.4)	
Servings of legumes per week (%)			<0.001
Never or rarely	2544 (11.3)	2468 (14.3)	
2 or 3 servings per week	14,055 (62.5)	10,107 (58.4)	
3 or more servings per week	5887 (26.2)	4735 (27.4)	
Servings of fish per week (%)			<0.001
Never or rarely	2335 (10.4)	2000 (11.6)	
2 or 3 servings per week	17,745 (78.9)	12,361 (71.4)	
3 or more servings per week	2413 (10.7)	2949 (17.0)	
Lifestyle			
Physical activity (h/week)			
Light physical activity	4.8 (4.7)	4.7 (4.0)	0.343
Moderate physical activity	3.7 (3.7)	3.0 (3.3)	<0.001
Intense physical activity	4.2 (4.1)	3.6 (3.2)	<0.001
Total physical activity	15.2 (10.2)	11.9 (8.5)	<0.001
Smoking status (%)			<0.001
Former	5940 (26.0)	3303 (19.1)	
Current	5348 (24.0)	3197 (18.4)	
Quality of Life Features			
In general, would you say your health is: (%)			<0.001
Poor/fair	7708 (34.0)	2916 (16.8)	
Good	10,873 (48.0)	9796 (56.6)	
Very good/excellent	3930 (18.0)	4613 (26.6)	

Participants over 18 years of age from the Spanish National Health Survey 2017 were included. HBP: High Blood Pressure, BMI: Body Mass Index; in characteristic sex, the category “other” has been excluded.

**Table 2:** Sociodemographic Data, Health Characteristics, Dietary Habits, Lifestyle and Quality of Life Features of the participants Categorized by Cardiometabolic Health Status (healthy/diseased).

Characteristics	Healthy Cardiometabolic Status	Diseased Cardiometabolic Status	*p*-Value
*n*(%)	12,303 (71.0)	5028 (29.0)	
Sociodemographic data			
Age (%)			<0.001
18–40 years	5699 (46.3)	1079 (21.5)	
40–70 years	6337 (51.5)	3439 (68.4)	
>70 years	267 (2.2)	510 (10.1)	
Sex (%)			<0.001
Male	4115 (33.5)	2287 (45.5)	
Female	8133 (66.1)	2729 (54.3)	
Education (%)			<0.001
High school or less	1774 (14.4)	1012 (20.1)	
More than high school	10529 (85.6)	4016 (79.9)	
Occupation (%)			<0.001
Unemployed/retired	1968 (15.8)	1381 (27.5)	
Worker	9251 (75.4)	3459 (68.8)	
Student	1084 (8.8)	188 (3.7)	
Anthropometric data			
BMI Category (WHO Criteria, %)			<0.001
Underweight (<18.5 kg/m^2^)	561 (4.6)	72 (1.4)	
Normal weight (18.5–24.9 kg/m^2^)	8445 (68.6)	1665 (33.1)	
Overweight (25.0–29.9 kg/m^2^)	3294 (26.8)	1444 (28.7)	
Obesity (>30 kg/m^2^)	3 (0.0)	1847 (36.7)	
Family history of cardiometabolic diseases			
Family history of obesity (%)	1924 (15.6)	1143 (22.7)	<0.001
Family history of diabetes (%)	3178 (25.8)	1837 (36.5)	<0.001
Family history of HBP (%)	5122 (41.6)	2819 (56.1)	<0.001
Family history of dyslipidemia (%)	4524 (36.8)	2520 (50.1)	<0.001
Dietary Habits			
Mediterranean Adherence Score	7.6 (2.1)	7.4 (2.2)	<0.001
Number of meals per day (%)			<0.001
1 or 2 meals	954 (7.8)	470 (9.3)	
3 meals	5396 (43.9)	2428 (48.3)	
4 meals	3822 (31.1)	1377 (27.4)	
5 or more meals	2127 (17.3)	752 (15.0)	
Snacking habit (%)	6036 (49.1)	2420 (48.1)	0.270
Servings of vegetables per day (%)			<0.001
1 or no serving per day	6797 (55.3)	2594 (51.7)	
2 or 3 servings per day	1519 (12.4)	473 (9.4)	
More than 3 servings per day	3973 (32.3)	1953 (38.9)	
Servings of legumes per day (%)			0.080
Never or rarely	8715 (70.9)	3645 (72.6)	
2 or 3 servings per week	2126 (17.3)	823 (16.4)	
More than 3 servings per week	1448 (11.8)	552 (11.0)	
Servings of fish per day (%)			<0.001
Never or rarely	7678 (62.5)	3124 (62.2)	
2 or 3 servings per week	2421 (19.7)	1107 (22.1)	
More than 3 servings per week	2190 (17.8)	789 (15.7)	
Lifestyle			
Nap habit (%)	3701 (30.1)	1973 (39.2)	<0.001
Physical activity (h/week)			
Light physical activity	4.7 (4.1)	4.7 (3.9)	0.004
Moderate physical activity	3.0 (3.3)	3.0 (3.3)	0.723
Intense physical activity	3.6 (3.2)	3.4 (3.2)	0.637
Total physical activity	12.0 (8.5)	11.7 (8.5)	0.075
Smoking status (%)			<0.001
Former	2036 (16.5)	1267 (25.2)	
Current	2170 (17.6)	1027 (20.4)	
Quality of Life Features			
In general, would you say your health is: (%)			<0.001
Poor/fair	1491 (12.1)	1424 (28.3)	
Good	6988 (56.8)	2808 (55.9)	
Very good/excellent	3821 (31.1)	792 (15.8)	
PCS12 (points)	54.5 (6.2)	51.0 (8.0)	<0.001
MCS12 (points)	43.5 (10.7)	44.7 (10.7)	<0.001

HBP: High Blood Pressure; BMI: Body Mass Index; MCS12: Mental Component Summary Score; PCS12: Physical Component Summary Score.

**Table 3:** Sociodemographic Data, Health Characteristics, Dietary Habits, Lifestyle and Quality of Life Features of the participants Categorized by Ethnicity (European/Caucasian and other ethnicities).

Characteristics	European/Caucasian	Other Ethnicities	*p*-Value
*n* (%)	11,233 (64.8)	5756 (33.2)	
Sociodemographic data			
Age (%)			<0.001
18–40 years	4267 (38.0)	2358 (41.0)	
40–70 years	6534 (58.2)	3062 (53.2)	
>70 years	432 (3.8)	336 (5.8)	
Sex (%)			<0.001
Male	3875 (34.5)	2411 (41.9)	
Female	7336 (65.3)	3330 (57.9)	
Education (%)			<0.001
High school or less	1475 (13.1)	1204 (20.9)	
More than high school	9758 (86.9)	4552 (79.1)	
Occupation (%)			<0.001
Unemployed/retired	1674 (14.9)	1224 (21.3)	
Worker	8818 (78.5)	4037 (70.1)	
Student	741 (6.6)	495 (8.6)	
Anthropometric data			
BMI Category (WHO Criteria, %)			<0.001
Underweight (<18.5 kg/m^2^)	426 (3.8)	184 (3.2)	
Normal weight (18.5–24.9 kg/m^2^)	6749 (60.1)	3165 (55.0)	
Overweight (25.0–29.9 kg/m^2^)	2947 (26.2)	1699 (29.5)	
Obesity (>30 kg/m^2^)	1110 (9.9)	708 (12.3)	
Cardiometabolic diseases prevalence			
Obesity (%)	1108 (9.9)	705 (12.2)	<0.001
Diabetes (%)	391 (3.5)	229 (4.0)	0.111
HBP (%)	1001 (8.9)	653 (11.3)	<0.001
Dyslipidemia (%)	1638 (14.6)	939 (16.3)	0.003
Family history of cardiometabolic diseases			
Family history of obesity (%)	1928 (17.2)	1099 (19.1)	<0.001
Family history of diabetes (%)	3089 (27.5)	1833 (31.8)	<0.001
Family history of HBP (%)	5192 (46.2)	2642 (45.9)	0.308
Family history of dyslipidemia (%)	4714 (42.0)	2219 (38.6)	<0.001
Dietary Habits			
Mediterranean Adherence Score	7.7 (2.1)	7.2 (2.6)	<0.001
Number of meals per day (%)			<0.001
1 or 2 meals	834 (7.4)	560 (9.7)	
3 meals	5040 (44.9)	2623 (45.6)	
4 meals	3463 (30.8)	1643 (28.5)	
5 or more meals	1892 (16.8)	929 (16.1)	
Snacking habit (%)	5214 (46.4)	3079 (53.5)	<0.001
Servings of vegetables per day (%)			<0.001
1 or no serving per day	3662 (32.6)	2160 (37.6)	
2 or 3 servings per day	6197 (55.2)	3019 (52.5)	
More than 3 servings per day	1361 (12.1)	572 (9.9)	
Servings of legumes per day (%)			<0.001
Never or rarely	1235 (11.0)	717 (12.5)	
2 or 3 servings per week	8180 (72.9)	3958 (68.8)	
More than 3 servings per week	1805 (16.1)	1076 (18.7)	
Servings of fish per day (%)			<0.001
Never or rarely	1696 (15.1)	1201 (20.9)	
2 or 3 servings per week	7075 (63.1)	3518 (61.2)	
More than 3 servings per week	2449 (21.8)	1032 (17.9)	
Lifestyle			
Nap habit (%)	3524 (31.4)	2042 (35.5)	<0.001
Physical activity (h/week)			
Light physical activity	4.8 (4.0)	4.7 (4.1)	0.153
Moderate physical activity	2.9 (3.2)	3.2 (3.4)	0.008
Intense physical activity	3.5 (3.0)	3.7 (3.4)	0.012
Total physical activity	11.8 (8.2)	12.2 (9.0)	0.023
Smoking status (%)			<0.001
Former	2196 (19.5)	1052 (18.3)	
Current	2143 (19.1)	977 (17.0)	
SF12 Health Survey			
In general, would you say your health is: (%)			<0.001
Poor/fair	1776 (15.8)	1077 (18.7)	
Good	6428 (57.3)	3167 (55.0)	
Very good/excellent	3027 (26.9)	1510 (26.3)	
MCS12 (points)	43.9 (10.6)	43.9 (10.9)	0.817
PCS12 (points)	53.7 (6.8)	53.0 (7.1)	<0.001

Other ethnicities include: Africans, Asians, Hispanic/Latinos, mestizos and other ethnicities. HBP: High Blood Pressure; BMI: Body Mass Index; MCS12: Mental Component Summary Score; PCS12: Physical Component Summary Score.

**Table 4:** Sociodemographic Data, Health Characteristics, Dietary Habits, Lifestyle and Quality of Life Features of the participants Categorized by Item 1 of the SF-12 Health Survey (“In general, would you say your health is?”).

Characteristics	Poor/Fair HRQoL	Good Health HRQoL	Very Good/ Excellent HRQoL	*p*-Value
*n*(%)	2916 (16.8)	9796 (56.5)	4613 (26.6)	
Sociodemographic data				
Age (%)				<0.001
18–40 years	1243 (42.6)	3626 (37.0)	1906 (41.3)	
40–70 years	1521 (52.2)	5713 (58.3)	2540 (55.1)	
>70 years	152 (5.2)	457 (4.7)	167 (3.6)	
Sex (%)				<0.001
Male	994 (34.1)	3581 (36.6)	1825 (39.6)	
Female	1913 (65.6)	6181 (63.1)	2764 (59.9)	
Education (%)				<0.001
High school or less	674 (23.1)	1498 (15.3)	610 (13.2)	
More than high school	2242 (76.9)	8298 (84.7)	4003 (86.8)	
Occupation (%)				<0.001
Unemployed/retired	749 (25.7)	1785 (16.2)	793 (17.2)	
Worker	1915 (65.7)	7373 (77.3)	3436 (74.5)	
Student	252 (8.6)	638 (6.5)	384 (8.3)	
Anthropometric data				
BMI Category (WHO Criteria, %)				<0.001
Underweight (<18.5 kg/m^2^)	83 (2.8)	327 (3.3)	223 (4.8)	
Normal weight (18.5–24.9 kg/m^2^)	1162 (39.9)	5669 (57.9)	3276 (71.0)	
Overweight (25.0–29.9 kg/m^2^)	936 (32.1)	2859 (29.2)	939 (20.4)	
Obesity (>30 kg/m^2^)	734 (25.2)	941 (9.6)	175 (3.8)	
Cardiometabolic diseases prevalence				
Obesity (%)	734 (25.2)	936 (9.6)	175 (3.8)	<0.001
Diabetes (%)	251 (8.6)	313 (3.2)	67 (1.5)	<0.001
HBP (%)	513 (17.6)	917 (9.4)	248 (5.4)	<0.001
Dyslipidemia (%)	676 (23.2)	1482 (15.1)	452 (9.8)	<0.001
Family history of cardiometabolic diseases				
Family history of obesity (%)	187 (6.4)	582 (5.9)	198 (4.3)	<0.001
Family history of diabetes (%)	140 (4.8)	429 (4.4)	188 (4.1)	<0.001
Family history of HBP (%)	253 (8.7)	790 (8.1)	313 (6.8)	<0.001
Family history of dyslipidemia (%)	332 (11.4)	1034 (10.6)	441 (9.6)	<0.001
Dietary Habits				
Mediterranean Adherence Diet Score	6.7 (2.1)	7.5 (2.1)	8.0 (2.1)	<0.001
Number of meals a day (%)				<0.001
1 or 2 meals	371 (12.7)	725 (7.4)	327 (7.1)	
3 meals	1329 (45.6)	4499 (46.0)	1994 (43.2)	
4 meals	803 (27.5)	2997 (30.6)	1396 (30.3)	
5 or more meals	413 (14.2)	1570 (16.0)	896 (19.4)	
Snacking habit (%)	1675 (57.4)	4745 (48.5)	2035 (44.1)	<0.001
Servings of vegetables per day (%)				<0.001
1 or no serving per day	1394 (47.9)	3389 (34.6)	1143 (24.8)	
2 or 3 servings per day	1325 (45.5)	5386 (55.0)	2681 (58.2)	
More than 3 servings per day	194 (6.7)	1015 (10.4)	783 (17.0)	
Servings of legumes per week (%)				<0.001
Never or rarely	484 (16.6)	1084 (11.1)	432 (9.4)	
2 or 3 servings per week	2033 (69.8)	7179 (73.3)	3149 (68.4)	
More than 3 servings per week	396 (13.6)	1527 (15.6)	1026 (22.3)	
Servings of fish per week (%)				<0.001
Never or rarely	726 (24.9)	1576 (16.1)	677 (14.7)	
2 or 3 servings per week	1727 (59.3)	6224 (63.6)	2852 (61.9)	
More than 3 servings per week	460 (15.8)	1990 (20.3)	1078 (23.4)	
Lifestyle				
Nap habit (%)	1025 (35.2)	3146 (32.1)	1502 (32.6)	0.009
Physical activity (h/week)				
Light physical activity	4.2 (4.0)	4.7 (4.0)	5.2 (4.2)	<0.001
Moderate physical activity	2.5 (3.1)	2.8 (3.1)	3.6 (3.6)	<0.001
Intense physical activity	2.9 (3.0)	3.3 (2.9)	4.4 (3.5)	<0.001
Total physical activity	10.4 (8.3)	11.3 (8.1)	13.5 (9.0)	<0.001
Smoking status (%)				<0.001
Former	575 (19.7)	1923 (19.6)	805 (17.5)	
Current	755 (25.9)	1807 (18.4)	631 (13.7)	
Quality of Life Features				
MCS12 (points)	39.2 (12.0)	43.7 (10.5)	46.8 (9.3)	<0.001
PCS12 (points)	45.2 (9.3)	54.2 (5.3)	56.9 (4.0)	<0.001

HBP: High Blood Pressure; BMI: Body Mass Index; MCS12: Mental Component Summary Score; PCS12: Physical Component Summary Score.

## Data Availability

Not required.
